# The dual role of sertraline: From antidepressant to antifungal drug with broad metabolic disruption

**DOI:** 10.1590/1678-4685-GMB-2025-0183

**Published:** 2026-04-03

**Authors:** Mayara I. G. Azevedo, Maria J. Santos-Saboya, João Neves-da-Rocha, Monise F. Petrucelli, Nilce M. Martinez-Rossi, Antonio Rossi

**Affiliations:** 1 Universidade de São Paulo, Faculdade de Medicina de Ribeirão Preto, Departamento de Genética, Ribeirão Preto, SP, Brazil.

**Keywords:** SSRI, drug repurposing, serotonin, alternative splicing, stress response

## Abstract

Sertraline, a selective serotonin reuptake inhibitor, is a widely used antidepressant. In recent decades, research has revealed its potential for drug repurposing, demonstrating various biological activities beyond psychiatric applications, including antifungal, antibacterial, antitumor, anthelmintic, and leishmanicidal effects. This review explores the antifungal potential of sertraline and its possible mechanisms of action against fungal cells, highlighting similarities with its effects in higher eukaryotes. We examine the effects of sertraline on (1) gene expression pathways, (2) lipid and carbohydrate metabolism, (3) membrane function, (4) stress response, and (5) alternative splicing modulation. Finally, we discuss future perspectives on the use of sertraline as an antifungal agent.

## Introduction

Serotonin (5-hydroxytryptamine) is a natural metabolite derived from the essential amino acid tryptophan that mediates signal transmission between nerve cells and other cells throughout the body. It is a neurotransmitter without hormonal properties. Serotonin regulates multiple functions in humans, including mood, libido, anxiety, cognitive functions, and various other sensitivities. It contributes to a more pleasant life and is popularly known as the “happiness hormone” (Shu et al., 2025). Serotonin is synthesized in the central nervous system, blood platelets, and enteric nervous system, with the assistance of specific bacteria that produce it. A significant proportion of serotonin is produced and stored in the cells of the gastrointestinal tract, from where it is distributed through the bloodstream (Guzel and Mirowska-Guzel 2022; Luo et al., 2024). The effect of serotonin in humans is concentration-dependent; both deficiency and excess can lead to mental disorders. A lack of serotonin may cause depression, low mood, and behavioral changes, among other effects. Excess serotonin, known as serotonin syndrome, may result in altered mental status and delirium (Volpi-Abadie et al., 2013; Shu *et al*., 2025).

**Figure 1:**
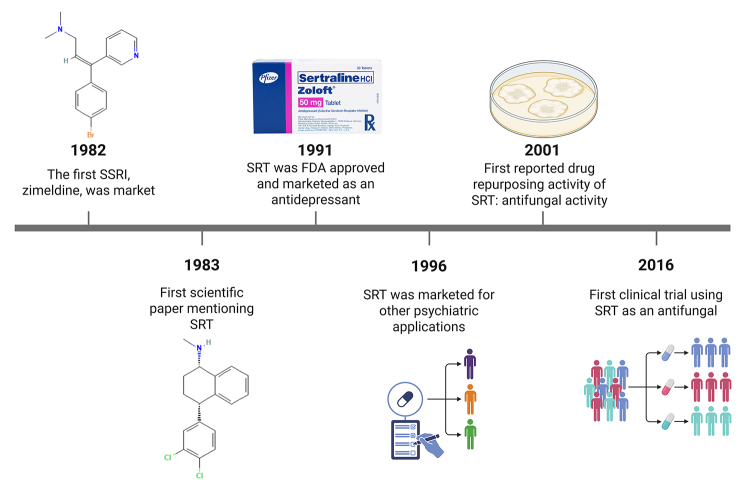
Timeline of sertraline use as an antidepressant and its drug repurposing. Starting from the left, the chemical structure of zimeldine, the first selective serotonin reuptake inhibitor (SSRI) to be marketed, is shown. Next, the chemical structure of sertraline is presented.

Selective serotonin reuptake inhibitors (SSRIs)-the first class of psychotropic medications developed through rational drug design-have been on the market since 1982 (Figure 1). They function by blocking the reabsorption of serotonin in the brain (Hillhouse and Porter 2015; Danilov 2015). Sertraline, an SSRI, is widely used to treat symptoms associated with various mental disorders such as depression, panic disorder, and anxiety, alleviating symptoms and promoting well-being. Its action involves increased serotonin levels in the brain, selective inhibition of serotonin reuptake, and elevated serotonin concentrations in synaptic clefts of neurons (Serretti et al., 2010). Disrupting this delicate balance of serotonin concentration-either increasing or decreasing it-can cause toxicity, including withdrawal symptoms or serotonin syndrome (Fava et al., 2015; Cooper et al., 2023).

A decade after its market release, sertraline was found to have also non-psychiatric applications, including antifungal activity (Figure 1). Patients with premenstrual dysphoric disorder (PMDD) and recurrent vulvovaginal candidiasis (VVC) were treated with sertraline for PMDD, and no recurrent episodes of acute VVC occurred during treatment (Lass-Flörl et al., 2001). This finding suggested that sertraline could be a promising candidate for drug repurposing (Baú-Carneiro et al., 2022). Drug repurposing strategies aim to identify new medical uses for approved or investigational drugs that are already considered safe for human use (Pushpakom et al., 2018). Due to its broad applicability and low cost, several drug repurposing applications have been documented for sertraline, including antifungal (Alanís-Ríos et al., 2025), antitumor (Jiang et al., 2018), antibacterial (Endo et al., 2025), anthelmintic (Weeks et al., 2018), and leishmanicidal (Ferreira et al., 2018) effects. 

Sertraline shows strong potential as a large-scale antifungal agent for human use (Table 1). This potential depends on a comprehensive understanding of its molecular mechanism of action across various species, including humans and fungi, as well as its roles in antidepressant activity, drug resistance, physiology, and responses to molecular signals. This understanding-supported by large-scale analytical techniques such as transcriptomics, proteomics, and other omics-is crucial for the safe application of sertraline as an antifungal drug or for determining its unsuitability. This review summarizes current knowledge on the antifungal properties of sertraline, highlighting its disruptive effects on fungal metabolism and potential parallels in higher eukaryotes.

**Table 1:** Sertraline activity against pathogenic fungi alone and in combination with antifungal agents.

Fungal species	Antifungal agents	Major findings	References
*Candida auris*	Voriconazole	This is the first preclinical study to evaluate the antifungal activity of sertraline, alone and in combination with an antifungal, against *C. auris*	Alanís-Ríos et al. (2025)
*C. albicans;* *C. kefyr;* *C. glabrata;* *C. krusei;* and *C. tropicalis*	*Cinnamomum verum* L. essential oil	The combination of sertraline and *Cinnamomum verum* L. essential oil showed significant synergism against planktonic cells and *Candida* biofilms	Barbarossa et al. (2024)
*Cryptococcus* spp. strains	Azoles	It has been suggested that the antifungal activity of sertraline and other selective serotonin reuptake inhibitors (SSRIs) is due to mitochondrial membrane damage and increased production of reactive oxygen species	Silva et al. (2023)
*Candida* spp. strains	Antifungals	It has been suggested that sertraline acts on *Candida* cells by causing cell wall and membrane damage	Rodrigues et al. (2023a)
*C. albicans;* *Candida krusei;* *and Candida glabrata*	Fluconazole and itraconazole	Sertraline was effective in reducing fungal biomass and metabolic activity	Ahmed et al. (2023)
*Trichophyton rubrum*	Caspofungin	The metabolic activity and biomass of the *T. rubrum* biofilm were influenced by sertraline alone and by its combination with caspofungin	Rocha et al. (2022)
*Cryptococcus neoformans*	None	Sertraline treatment induces supersized lipid droplets	Breuer et al. (2022)
*Candida auris*	None	It has been suggested that Sertraline does not affect the cell wall but instead acts by binding to the sterol 14-α-demethylase, an enzyme involved in ergosterol biosynthesis	Gowri et al. (2020)
*Sporothrix schenckii* strains	Itraconazole, voriconazole and amphotericin B	Sertraline demonstrated synergistic effects with itraconazole in one strain, primarily additive effects with voriconazole, and indifferent effects with amphotericin B	Villanueva-Lozano et al. (2019)
*Aspergillus fumigatus*	None	Sertraline treatment improved larval survival and health index scores of *Galleria mellonella,* and reduced pulmonary fungal burden in a murine model of invasive pulmonary aspergillosis	Treviño-Rangel et al. (2019)
*Trichosporon asahii* isolates	Fluconazole, voriconazole, itraconazole, caspofungin and amphotericin B	Sertraline displayed synergistic effects against *T. asahii* planktonic cells when combined with amphotericin B, caspofungin, or fluconazole, and also showed synergistic effects against *T. asahii* biofilms in combination with amphotericin B	Cong et al., (2016)

## From DNA to protein: The multifaceted influence of sertraline 

### Translation

**Figure 2:**
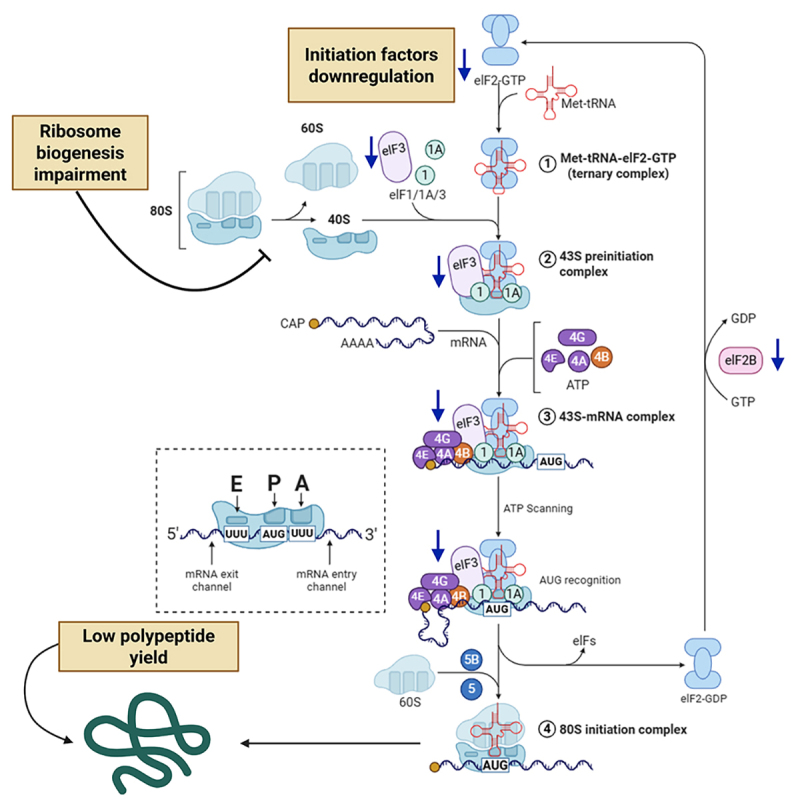
Effects of sertraline on translation. Sertraline’s reported impacts on several steps of translation in both fungi and higher eukaryotes. Blue arrows indicate downregulated genes encoding translation factors. The black arrows indicate effects observed in both higher eukaryotes and fungi, supported by experimental evidence. The blue arrows indicate RNA-seq data.

Several reports have been published on the impact of sertraline on cell signaling pathways in humans, ranging from immunomodulatory effects to the suppression of cytokine levels and the inhibition of translation initiation via downregulation of the mTOR pathway (Lin et al., 2010; Önal *et al*., 2025). Similar to humans, translation initiation is perturbed in fungi (Figure 2). A study of the mechanism of action of sertraline using a genome deletion collection of *Saccharomyces cerevisiae* with altered sertraline susceptibility found that the most sensitive mutants had a disrupted translation initiation factor, Tif3. The effect of sertraline on *Cryptococcus neoformans* is also related to a disturbance in translation initiation, affecting polypeptides yield in a dose-dependent manner (Zhai et al., 2012). In *Trichophyton rubrum*, translation is disrupted by the downregulation of many proteins involved in ribosome biogenesis, assembly, and pre-rRNA processing (Rrp14, MAK16, loc1, Urb1, ERB1, RPF2, SQT1, RIX7, Srp40, Brx1, and Gar2). Additionally, sertraline treatment downregulates numerous genes encoding translation initiation factors, predominantly eIF3 subunits as well as eIF2, eIF4, and eIF5 (Galvão-Rocha et al., 2023) (Figure 2).

Ribosome biogenesis initiates in the nucleus with the transcription and processing of ribosomal RNA, followed by the recruitment of ribosomal proteins. The partially assembled ribosome is subsequently exported to the cytoplasm where it undergoes final assembly and maturation. Disruption of any stage of this tightly regulated process-including rRNA synthesis, ribosomal protein incorporation, or nucleocytoplasmic transport-can compromise nuclear integrity. Such defects often trigger cell cycle arrest and prevent aberrant cell division, thereby maintaining genomic stability (Turi et al., 2019). For example, Rrp14 facilitates the nuclear translocation of Pol5, which directly influences the recycling and transport of 60S and small ribosomal subunits. Gar2 is involved in assembling of the 40S subunit (Sicard et al., 1998; Lin et al., 2022). 

Initiation-the most intricate and regulated phase of translation-is profoundly influenced by the multifaceted role of eIF3 (Figure 2). Eukaryotes possess approximately 11 translation initiation factors, many of which have several subunits, and rely on eIF3 for essential function. These include recruit the ribosome to mRNA, stabilizing eIF2 on the ribosome, and linking ribosome recycling to the initiation of a new translation cycle (Brito et al., 2024; Liu et al., 2025b). In humans, sertraline-induced inhibition of translation is associated with decreased levels of the eIF4 complex, increased nuclear retention, and phosphorylation of eIF2α. Like eIF3, eIF4 is critical for ribosomal recruitment, highlighting their interdependence. eIF2 forms a ternary complex (eIF2-GTP-tRNAi^Met) and mediates start codon recognition (Lin et al., 2010). Taken together, the available evidence supports the hypothesis that sertraline directly affects eukaryotic translation, from ribosomal biogenesis to polypeptide chains synthesis. Figure 2 summarizes sertraline’s effects on translation. 

### Transcription

Transcription is a highly coordinated process involving multiple steps and regulatory proteins, including transcription factors (TFs). In *T. rubrum*, sertraline treatment significantly alters TF gene expression patterns, upregulating stress-responsive TFs and downregulating those involved in cellular differentiation, homeostasis, and cell cycle control. Sertraline also upregulates autophagy-related genes in *T. rubrum*, mirroring observations in human cells, where sertraline induces nuclear translocation of transcription factor EB (TFEB), a master regulator of lysosomal biogenesis and autophagy. In humans, this TFEB-mediated response is linked to sertraline-induced cholesterol accumulation in lysosomes, which subsequently triggers the activation of autophagy pathways (Galvão-Rocha et al., 2023; Alvarez-Valadez et al., 2024). Although autophagy is essential for regulating the turnover of macromolecules and organelles, its dysregulation is associated with type II autophagic cell death and various human diseases (Khandia et al., 2019; Sehrawat et al., 2023).

To date, no studies have reported sertraline-induced transcriptional impairments in other fungal species. This knowledge gap may stem from the predominant focus of existing research on the broad-spectrum antifungal effects of sertraline, such as metabolic inhibition, biomass reduction, and reactive oxygen species (ROS) generation (Table 1). Further investigations employing transcriptomic or proteomic approaches, similar to the aforementioned study on *T. rubrum*, are required to elucidate the full extent of influence of sertraline on gene expression and other underexplored cellular processes.

### Replication

DNA replication is a highly regulated process that requires the precise coordination of multiple enzymatic activities. Effective DNA damage response mechanisms are essential for maintaining genomic integrity and preventing deleterious mutations (Steenwyk 2021). In *T. rubrum*, sertraline treatment downregulates key DNA repair enzymes, including RAD26, MSH6, and UvdE, potentially compromising replication fidelity. Although these findings suggest that sertraline may interfere with DNA maintenance processes, no studies have examined its effects on replication or DNA damage responses in other fungal species (Galvão-Rocha et al., 2023). Basic assays, such as comet assay, could provide valuable preliminary data to address this knowledge gap. However, the potential genotoxicity of sertraline in other eukaryotes remains controversial. One study reported no detectable DNA damage in albino rats following sertraline exposure (Battal et al., 2013), whereas studies in *Drosophila melanogaster* suggest that sertraline induces DNA double-strand breaks in mitotically active tissues and significantly increases the frequency of apoptosis (Jajoo et al., 2020). The genotoxic effects of sertraline appear to exhibit species-specificity, in contrast to its conserved impact on translation across eukaryotes. This selective DNA damage profile suggests the potential for drug repurposing, as it may enable targeted antifungal activity while minimizing host toxicity. However, comprehensive studies across diverse eukaryotic models are required to validate this specificity and establish a mechanistic basis.

## Sertraline’s effect on metabolism

### Mitochondrial metabolism

Energy metabolism is a complex network of biochemical pathways that convert nutrients into adenosine triphosphate (ATP), the primary energy source for living organisms. This process is meticulously regulated, and any imbalance is often associated with mitochondrial disorders (Liu et al., 2025a). Sertraline perturbs *T. rubrum* mitochondria by downregulating alternative oxidase (Aox) and isocitrate dehydrogenase (IDH). Aox is an enzyme involved in the reduction of ROS generated in the respiratory chain, whereas IDH is a regulatory enzyme of the citric acid cycle. The downregulation of both enzymes may significantly impair mitochondrial function and cellular energy production (Edrich et al., 2024; Shi et al., 2025). In addition, an intriguing effect of sertraline on *T. rubrum* mitochondria is the upregulation of Complex I and several proteins involved in ubiquinone biosynthesis. This effect is likely due to sertraline-induced inhibition of the respiratory complex. Inhibition of mitochondrial complexes has also been reported in humans and rats. Sertraline induces mitochondrial dysfunction in human astrocytes, rat hepatocytes, and brain cells (Kumar and Kumar 2009; Li *et al*., 2012; Then et al., 2017). Case reports further indicate that mitochondrial impairment by sertraline may cause muscle weakness and multiple acyl-CoA dehydrogenase deficiencies in treated patients. Most of these studies show that sertraline impairs the respiratory chain by disrupting the activity of Complexes I, II, and IV (Hedberg-Oldfors et al., 2024; Ingoglia et al., 2024). The effect on mitochondria has also been reported in other fungi, such as *Cryptococcus* spp. In this study, the authors attributed sertraline’s antifungal activity to its ability to disrupt the mitochondrial membrane (Silva et al., 2023). These findings highlight the impact of sertraline on mitochondria and, consequently, on energy metabolism in both fungi and higher eukaryotes, underscoring the broader implications of this research for mitochondrial biology.

### Lipid and carbohydrate metabolism

Lipid metabolism is essential for cellular homeostasis, serving as a key structural component of membranes and playing crucial roles in signaling and nutrient storage. Previous studies have demonstrated that sertraline disrupts lipid metabolism in diverse fungal species, including *C. neoformans*, *Candida albicans*, *S. cerevisiae*, and *Aspergillus fumigatus*, by promoting the formation of supersized lipid droplets (SLDs) (Breuer et al., 2022). These SLDs resemble lipid storage myopathies, disorders characterized by pathological lipid accumulation in muscle fibers, which have been observed in humans following sertraline treatment (Hedberg-Oldfors et al., 2024). Further evidence of sertraline-induced disruption of fungal lipid metabolism includes its inhibitory activity on phosphatidic acid phosphatase (PAP) in *S. cerevisiae*. This central enzyme converts phosphatidic acid (PA) into diacylglycerol (DAG), thereby regulating phospholipid and triacylglycerol synthesis. Beyond fungi, this mechanism may extend to mammals, as sertraline’s structural similarity to PA suggests potential cross-kingdom inhibition of lipin1, a human PAP homolog (Stukey et al., 2025). Supporting this, Bozdag et al. (2024) demonstrated that sertraline disrupts adipogenesis in human adipocytes by upregulating phospholipid biosynthesis and lysosomal lipid processing, phenotypes indicative of impaired PA/DAG homeostasis (Bozdag *et al*., 2024). These similarities suggest a conserved mechanism of action for sertraline across eukaryotes, potentially linking its antifungal effects to broader metabolic dysregulation.

Furthermore, gluconeogenesis was downregulated in *T. rubrum* following sertraline treatment. Expression of two of the three key enzymes regulating this pathway was affected: two phosphoenolpyruvate carboxykinase genes and one fructose-1,6-bisphosphatase gene. Gluconeogenesis is a metabolic pathway that transforms non-sugar molecules into free glucose. In the context of sertraline-induced mitochondrial dysfunction, gluconeogenesis could serve as an alternative ATP-generating pathway by converting amino acids and pyruvate into glucose, which then feeds into glycolysis (Sunny et al., 2011). The data presented highlight the profound impact of sertraline on the metabolism of fungi and other eukaryotes.

### Membrane structure impairment caused by sertraline

The exact antifungal mechanism of sertraline remains unclear and may vary by species. However, it appears to differ from that of traditional antifungals, such as azoles and echinocandins, which respectively target ergosterol synthesis and the synthesis of β-glucans and other cell wall components, including chitin. Gowri et al. (2020) demonstrated that sertraline neither binds directly to ergosterol in the fungal membrane nor affects cell wall integrity, as evidenced by sorbitol protection and ergosterol supplementation assays. *In silico* docking analyses revealed that sertraline interacts with sterol 14-α-demethylase, a key enzyme in the ergosterol biosynthesis pathway. Inhibition of this enzyme resulted in a 5.5-fold reduction in ergosterol production in *Candida auris* (Gowri *et al*., 2020). Similarly, high-throughput transcriptome analysis by Galvão-Rocha et al. (2023) showed that sertraline downregulated multiple genes involved in ergosterol biosynthesis in *T. rubrum*. These genes encode diphosphomevalonate decarboxylase (*erg19*), C-8 sterol isomerase *(erg1*), C-14 sterol reductase (*erg24*), C-4 methylsterol oxidase (*erg25*), ergosterol biosynthesis protein (*erg28*), sterol 24-C-methyltransferase (*erg6*), and squalene epoxidase (*erg1*). Consistent with these findings, a significant reduction in ergosterol levels was observed upon exposure to sertraline (Galvão-Rocha *et al*., 2023).

**Figure 3:**
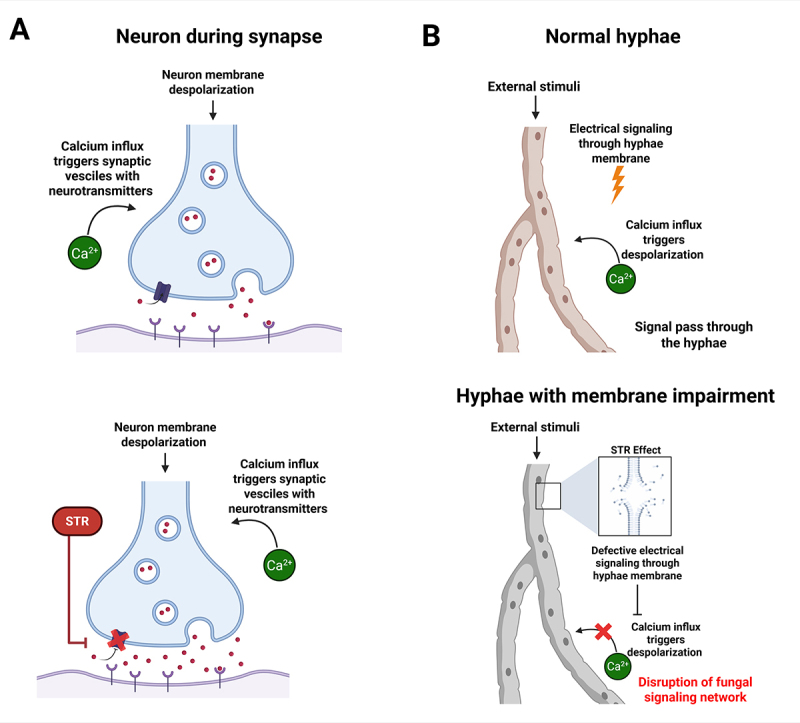
Comparison of neuronal and hyphal communications, and how membrane impairment affects fungal signaling. (A) represents both the normal neuron synapse (left side) and the effect of sertraline on this process (right side). (B) represents hyphal communications through cellular membrane electrical signaling (left side), and the disruption caused by sertraline-induced membrane impairment (right side). Both neurons and hyphae use calcium influx to trigger these processes. This image illustrates a proposed mechanism by which sertraline could affect hyphal communication.

The mechanism of action of sertraline in the mammalian nervous system is well established, primarily through the inhibition of the 5-hydroxytryptamine transporter and subsequent blockade of serotonin reuptake into presynaptic cells (Lu et al., 2024). No conserved homolog of the 5-hydroxytryptamine transporter exists in fungi. Several studies have suggested that sertraline exerts membrane-disruptive effects due to its amphipathic nature, allowing it to intercalate into the phospholipid bilayer and alter fungal membranes fluidity. In *S. cerevisiae*, sertraline has been shown to target intracellular vesiculogenic membranes. At concentrations of 120 µM, it induces phospholipidosis, characterized by the formation of multilamellar bodies and increased autophagy, potentially reflecting impaired membrane turnover and reduced digestibility by phospholipases (Rainey et al., 2010). Other studies have shown that sertraline compromises membrane permeability and inhibits sphingolipid biosynthesis, which is crucial for maintaining fungal membrane stability (Spitzer et al., 2011). These findings raise important questions about the molecular mechanisms underlying sertraline’s effects on fungal membranes and whether they parallel its known actions in human cells. Although fungi lack serotonin transporters, their membranes facilitate electrical signaling in response to external stimuli. For example, Ca^2+^ inflow at the hyphal tip influences actin depolymerization timing, a process functionally analogous to neuronal communication (Money 2021; Itani et al., 2023). Therefore, sertraline-induced membrane perturbations could disrupt fungal signaling networks, impairing the organism’s ability to sense and adapt to environmental changes (Hammadeh et al., 2022). These data suggest a potential convergence in sertraline’s mode of action across kingdoms, in which membrane-mediated signaling interference may underlie both antifungal and neuroactive effects (Figure 3). Taken together, these findings suggest that the antifungal activity of sertraline involves its interaction with the fungal plasma membrane lipid bilayer, leading to structural alterations that ultimately contribute to its fungicidal effects. 

## Sertraline-induced stress response 

### Cellular stress responses in fungi

Fungi adapt to diverse environmental conditions. Whether pathogenic or not, these organisms employ a range of mechanisms to cope with and survive in hostile environments. Cellular exposure to pharmacological agents often disrupts homeostasis, triggering oxidative stress, endoplasmic reticulum (ER) stress, accumulation of misfolded proteins, heat shock stress, and membrane disturbances (Brown et al., 2014; Persinoti et al., 2014; Chen et al., 2014; Mendes et al., 2018; Peres et al., 2022; Rocha et al., 2022).

Fungal cells undergoing ER stress activate the unfolded protein response, primarily via the Ire1-Hac1 signaling pathway, to restore proteostasis under protein-folding load. Failure to maintain this balance may impair virulence in species such as *A. fumigatus, T. rubrum,* and *C. albicans* (Askew 2014; Bitencourt et al., 2020). Additionally, the oxidative stress response is mediated by three highly conserved pathways among fungal species. The major modulators of these pathways are the high-osmolarity glycerol pathway, yeast-activating protein 1-like (Yap1) basic-leucine zipper (bZIP)-containing TF, and the response regulator and transcription factor Skn7 (Yaakoub et al., 2022). Despite significant niche-driven variations in stress sensitivity among fungal species, a landmark comparative study using multiple fungi revealed a striking conservation of the core components of osmotic, oxidative, and cell wall stress responses-facilitated by the identification of orthologous genes-underscoring the value of phylogenetic inference in investigations of cellular stress mechanisms (Nikolaou et al., 2009). Studying drug-induced stress benefits from framing responses at the cellular level, which involves identifying affected organelles (such as mitochondria, ER, and vacuoles), monitoring ROS or membrane integrity, and profiling stress-response genes (including heat shock proteins and antioxidant enzymes). These phenotypic and molecular markers provide a coherent framework for assessing the antifungal activity of sertraline.

### Sertraline-induced stress 

Antifungal drugs such as azoles and echinocandins have limited efficacy. For instance, fluconazole is fungistatic rather than fungicidal, whereas echinocandins are effective against *Aspergillus* and *Candida* species but ineffective against *Cryptococcus* (Zhai et al., 2012). These antifungals typically exploit fungal vulnerabilities by targeting ergosterol biosynthesis or cell wall synthesis, resulting in secondary stresses such as membrane destabilization and the accumulation of ROS.

As mentioned previously, in *T. rubrum*, sertraline downregulates ergosterol and membrane biosynthesis genes while upregulating stress-response transcripts, as revealed by RNA-seq profiling. This suggests the activation of oxidative detoxification and metabolic disruption (Galvão-Rocha et al., 2023). When *T. rubrum* is treated with undecanoic acid-a fatty acid with diverse cellular effects that causes fungal toxicity-several stress-response pathways and antioxidant enzymes are upregulated (Mendes et al., 2018; Rossi et al., 2021). These findings support the hypothesis that sertraline triggers multiorganelle stress in fungi. Moreover, its synergistic effect with caspofungin in *T. rubrum* indicates that sertraline exacerbates cell wall and membrane stress, overwhelming fungal compensatory mechanisms (Rocha et al., 2022). 

Studies on *C. auris* corroborate these findings: sertraline inhibits biofilm formation and disrupts membrane integrity. Recent in vivo studies further demonstrated that sertraline reduces fungal burden in a dose-dependent manner and exhibits synergistic effects with voriconazole (Gowri et al., 2020; Alanís-Ríos et al., 2025). This concentration-dependent response aligns with observations in mammalian systems, where increasing sertraline doses elevate oxidative stress markers, including lipid peroxidation (Battal et al., 2014), indicating that higher drug levels exacerbate cellular stress in eukaryotes.

Sertraline-induced stress extends to other organisms. In *Drosophila melanogaster*, the drug causes DNA double-strand breaks and cellular toxicity, which are mitigated by antioxidant treatment (e.g., ascorbic acid), indicating oxidative DNA damage (Jajoo et al., 2020). Although derived from an animal model, these findings suggest potential parallels in fungi. For example, *C. glabrata* upregulates glutathione-dependent antioxidant defenses in response to oxidative stress (Gutiérrez-Escobedo et al., 2013) and may employ base excision repair to counteract DNA damage (Yasui 2013).

Marine rotifers (*Brachionus koreanus*) exposed to sertraline or fluoxetine showed elevated ROS levels, growth inhibition, and increased catalase and Fe-superoxide dismutase (SOD) activity (Byeon et al., 2020). Similarly, fungal pathogens such as *C. neoformans* and *C. albicans* enhance antioxidant defenses (including glutathione, thioredoxin, catalase, and SOD) to mitigate ROS and sustain viability, even when primary redox systems are compromised (Cox et al., 2003; Missall and Lodge 2005; Black et al., 2024). Unlike higher eukaryotes, fungi possess distinct membrane compositions (ergosterol vs. cholesterol) and fewer redundant stress-response pathways. Their metabolic and detoxification genes often lack redundancy, potentially increasing their susceptibility to the disruptive effects of sertraline (Coelho et al., 2014; Galvão-Rocha et al., 2023).

### Fungal defense mechanism against sertraline

Upon exposure to sertraline, fungi rapidly activate canonical oxidative stress defense mechanisms. In *T. rubrum*, glutathione S-transferase (GST) genes are upregulated within 3 h of treatment, indicating an initial attempt at detoxification through glutathione conjugation and vacuolar extrusion. However, after 12 h, the expression of GSTs, SOD, and catalase declines, indicating a progressive collapse of antioxidant defenses (Galvão-Rocha et al., 2023). The thioredoxin/thioredoxin reductase (Trx/TrxR) system is similarly compromised downregulating TrxR while transiently upregulating Trx during prolonged exposure, thereby impairing redox homeostasis (Galvão-Rocha *et al*., 2023). Given the essential role of TrxR in *A. fumigatus* and *C. neoformans* and its structural divergence from mammalian homologs, this system represents a promising antifungal target (Missall and Lodge 2005; Leal et al., 2013).

Beyond redox systems, lipid metabolism plays a critical role in fungal drug resistance, biofilm formation, and the release of extracellular vesicles. Altered lipid composition and macrodomain organization can attenuate virulence and disrupt stress adaptation (Rella et al., 2016). Supersized lipid droplets observed in *C. neoformans*, *C. albicans*, *S. cerevisiae*, and *A. fumigatus* under sertraline exposure suggest a conserved membrane stress response, potentially reflecting impaired lipid catabolism or compensatory membrane remodeling (Breuer et al., 2022). Fungi also use drug efflux pumps to limit the intercellular accumulation of sertraline. In *Candida* biofilms, transporter overexpression diminishes drug efficacy, and efflux pump upregulation partially counteracts sertraline-induced biofilm inhibition. This defense mechanism is further exacerbated in mature biofilms, where extracellular matrix barriers significantly reduce drug penetration (Rodrigues et al., 2023b).

At the organelle level, autophagy and vacuolar sequestration mitigate sertraline toxicity. The drug accumulates in acidic vesicular membranes and induces autophagy to compartmentalize cytotoxic compounds, shield organelles, and generate protective lipid droplets (Rainey et al., 2010; Chen et al., 2012). These adaptations reflect systemic reprogramming of degradation and trafficking pathways to maintain homeostasis.

Cell wall and membrane remodeling are pivotal compensatory strategies. *Trichosporon asahii* upregulates chitin and β-glucan synthases to reinforce cell wall integrity under sertraline stress (Cong et al., 2016), whereas *Candida* spp. modulate ergosterol biosynthesis and lipid composition to stabilize membranes (Zhai et al., 2012). These modifications buffer the disruptive effects of sertraline, underscoring the importance of targeting adaptive pathways to enhance antifungal effectiveness.

### Sertraline modulates alternative splicing

Alternative splicing (AS) is a post-transcriptional regulatory mechanism that enhances transcriptomic and proteomic complexity in eukaryotic organisms (Grutzmann et al., 2014). Growing evidence indicates that AS plays an important role in the regulation of fungal gene expression, particularly under stress or changing environmental conditions (Muzafar et al., 2021). AS contributes to the dynamic regulation of gene expression by producing multiple mRNA isoforms from a single gene, which can lead to the production of functionally distinct proteins or in the regulation of mRNA stability and localization (Ule and Blencowe 2019; Marasco and Kornblihtt 2022).

The extensive occurrence of intron retention (IR) events in fungal transcriptomes suggests a regulatory rather than a splicing-defective mechanism. One of the most frequent effects of IR is the introduction of premature termination codons (PTCs), which can trigger mRNA degradation via the nonsense-mediated decay (NMD) pathway (Kurosaki et al., 2019; García-Moreno et al., 2020; Gao et al., 2022). Since AS and NMD are functionally associated processes, cells can use IR events to rapidly adjust mRNA levels in response to external stimuli or physiological cues, including antifungal drugs. However, the possibility that PTC generates a smaller yet functional protein or even a regulatory RNA cannot be excluded. In summary, AS represents an effective and reversible mechanism to modulate gene expression through the regulatory potential of the spliceosome machinery (Neves-da-Rocha et al., 2019; Gao *et al*., 2022). This mechanism may be particularly important when fungi are exposed to xenobiotic substances, such as sertraline.

### AS in fungi in response to sertraline exposure 

In pathogenic fungi, AS is linked to the regulation of genes involved in biological processes related to virulence, metabolism, adaptive strategies, and stress responses (Grutzmann et al., 2014; Mendes et al., 2018). A meta-analysis of the pathogens *Histoplasma capsulatum*, *A. fumigatus*, *C. albicans*, and *C. neoformans* revealed condition-dependent modulation of splicing under both host and stress conditions, indicating that AS has an adaptive function in regulating pathogenicity and survival (Sieber et al., 2018). Recent research on the dermatophyte *T. rubrum* revealed a global landscape of AS upon exposure to sertraline (Rocha et al., 2025). In this transcriptomic investigation, sertraline treatment modulated a significant number of AS events in *T. rubrum*, with IR being the most common AS type. Other AS types, such as alternative splice sites and exon skipping, were also detected but at much lower frequencies-a pattern already described in previous studies on filamentous fungi (Grutzmann *et al*., 2014). RNA-seq analysis of *T. rubrum* identified 351 AS events in 289 genes after 3 h of sertraline exposure and 1,051 AS events in 690 genes after 12 h. Furthermore, 145 of these genes showed no time dependence and were modulated at both time point. Transcriptome analysis revealed 43 genes that exhibited differential expression and AS events simultaneously after 3 and 12 h of sertraline treatment. The authors highlighted a peculiar expression pattern in genes that were simultaneously modulated: induction of differential expression was associated with downregulation of AS events, whereas repression was associated with their upregulation. This opposing modulatory pattern of expression and AS levels suggests that splicing serves as an additional regulatory layer, optimizing the use of conventional mRNA isoforms when specific genes are required. In contrast, when gene expression is no longer needed, AS may regulate mRNA turnover through IR coupled with NMD degradation. This trend was particularly evident in genes involved in key biological processes such as multidrug resistance proteins, transcription factors, and one gene involved in eukaryotic translation initiation (Rocha *et al*., 2025). IR events were particularly enriched in protein kinase genes, linking splicing modulation to fungal stress response pathways. AS affects both metabolic and regulatory genes, as well as known antifungal targets, indicating a broad regulatory network rather than target-specific effects. These observations are consistent with research in other pathogenic fungi, which show global shifts in splicing patterns in response to antifungal exposure, temperature changes, or nutrient deprivation (Grutzmann *et al*., 2014; Muzafar et al., 2021). Although the exact functional implications of AS modulation remain unclear understood, the occurrence of conserved AS patterns in response to various stressors highlights the importance of AS as a regulatory layer in fungal adaptation and survival-particularly in the context of drug resistance (Grutzmann *et al*., 2014; Sieber *et al*., 2018; Muzafar *et al*., 2021).

### AS and sertraline in higher eukaryotes

Although AS has distinct features in fungi and higher eukaryotes (Grutzmann et al., 2014), evidence suggests that sertraline’s potential to influence AS is not restricted to fungal cells. Recent studies using animal model species, including *D. melanogaster* and rats (Yamada et al., 1999; Santos-Cruz et al., 2025), have shown that chronic sertraline use affects AS in genes involved in neuronal function, cellular homeostasis, and stress responses, suggesting a conserved molecular signature of SSRI activity across phylogenetically distant organisms. The long-term effects of sertraline exposure during larval development on the splicing profiles of adult flies were investigated in *D. melanogaster* (Santos-Cruz et al., 2025). Significant alterations in AS profiles, including exon inclusion/exclusion and IR events, were found in central nervous system tissues analyzed using RNA-seq. Genes involved in neuronal function displayed altered AS: sertraline treatment led to the inclusion of long isoforms of *Ank2*, a gene associated with axonal stability, and of ATPalpha, a Na⁺/K⁺-ATPase subunit that maintains ionic gradients and neuronal excitability. In addition, IR was observed in the *Yuri*, a gene involved in cytoskeletal reorganization, suggesting that sertraline may also affect neuronal morphology. Other genes affected by splicing changes include *sxc*, a regulator of epigenetic states via O-GlcNAcylation, and *Atg18a*, which is involved in autophagy (Santos-Cruz *et al*., 2025).

In the rat frontal cortex, a second study in mammals identified a novel splice variant of the 70-kDa heat shock cognate protein following long-term antidepressant treatment (HSC70), termed HSC49. Due to the absence of exons 7 and 8, the HSC49 transcript encodes a truncated protein of approximately 48.6 kDa. Expression of this variant is markedly elevated at the mRNA and protein levels by imipramine and sertraline (Yamada et al., 1999). HSC70, a member of the HSP70 family, plays a crucial role in stress response and protein folding, which are essential in neurons and other cell types. The consistent appearance of AS changes upon sertraline exposure across phylogenetically distant organisms suggests that sertraline may act through conserved stress response pathways or directly influence spliceosomes or splicing factors, despite the absence of serotonin receptors or transporters in fungi. Understanding how sertraline affects AS across various systems could shed light on the drug’s pleiotropic effects across species and potentially reveal novel antifungal mechanisms. Sertraline may induce membrane disruption or stress signaling pathways, which subsequently affect the splicing machinery. Despite differences in cellular contexts, AS may represent a common target or readout of sertraline action, as indicated by the cross-species convergence of AS modulation under sertraline exposure.

## Future perspectives

**Figure 4:**
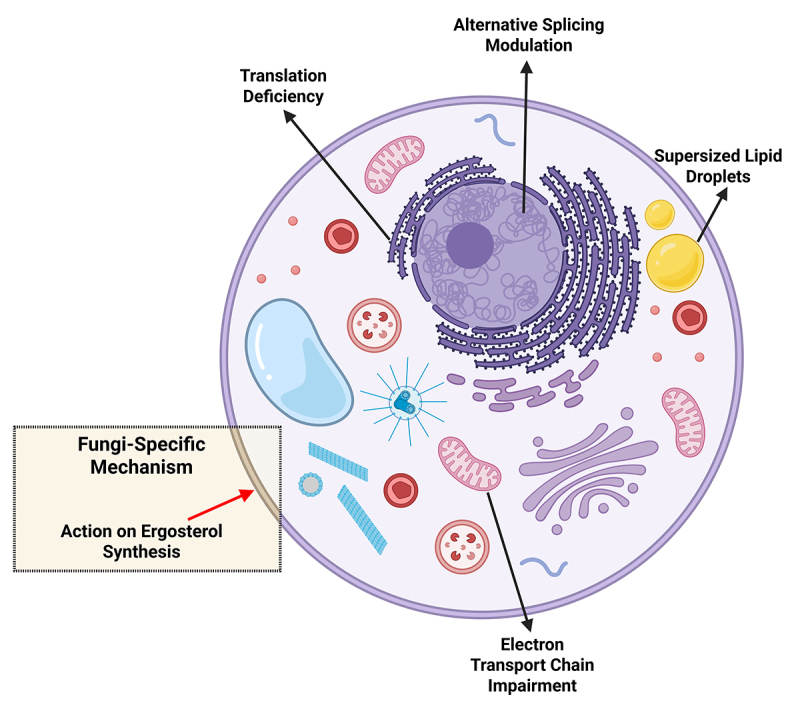
Cellular processes and metabolic pathways affected by sertraline. The figure represents a generic eukaryotic cell. Black arrows represent effects observed in fungi and other eukaryotes: translation deficiency, alternative splicing modulation, supersized lipid droplets, and electron transport chain impairment. Red arrow represents the effect observed only in fungi: inhibition of ergosterol synthesis.

The antifungal properties of sertraline were first identified over two decades ago, marking one of the earliest examples of this antidepressant’s potential for drug repurposing (Lass-Flörl et al., 2001). Subsequent studies have consistently demonstrated sertraline’s broad-spectrum activity against diverse fungal pathogens (Table 1). However, as highlighted in this review, sertraline’s mechanism of action exhibits cross-kingdom effects that extend to human cells (Figure 4). This characteristic raises critical therapeutic considerations about whether sertraline’s antifungal efficacy could potentially be achieved at concentrations that avoid significant toxicity in humans. Clinical trials to date have yielded discouraging results. In a study of HIV-associated cryptococcal meningitis, sertraline failed to reduce mortality. In another trial, severe adverse effects (including psychosis, aggressive behavior, and serotonin syndrome) were reported at doses of 200 mg/day, and the trial had to be stopped early without determining the efficacy of sertraline treatment against cryptococcal antigenemia (Rhein et al., 2019; Boulware et al., 2020). These findings highlight the challenge of harnessing sertraline’s antifungal properties while minimizing its off-target effects in eukaryotic hosts. 

A potential solution to this challenge could lie in the topical application of sertraline or its synergistic use with established antifungals to enhance efficacy. Sertraline has been shown to potentiate the activity of azoles and echinocandins against several fungal strains (Rocha et al., 2022; Rodrigues et al., 2023a; Alanís-Ríos et al., 2025). However, the clinical translation of these combinations requires careful evaluation, as even targeted antifungals, such as azoles, can induce off-target effects in human cells (Draskau and Svingen 2022). Further research is needed to determine whether sertraline-antifungal combinations can achieve therapeutic synergy at sub-toxic concentrations, thereby balancing efficacy and safety. Although topical sertraline formulations have not yet been evaluated for antifungal applications in clinical or preclinical studies, existing FDA-approved gel-based and transdermal delivery systems for psychiatric indications (Vijaya and Ruckmani 2011; Tijani et al., 2021) present immediately testable platforms. Given sertraline’s established anti-dermatophytic activity against *T. rubrum*, repurposing existing commercial formulations for topical application represents a promising strategy for dermatophytosis treatment. 

## Conclusion

As extensively shown in this review, sertraline presents a well-established antifungal activity. However, most of the mechanisms of action of this drug against fungi are shared with higher eukaryotes, affecting translation, transcription, energy metabolism, alternative splicing regulation, and stress pathways. Sertraline’s antifungal potential could be explored, focusing either on its effect on ergosterol synthesis, as higher eukaryotes do not have this sterol in their membranes, or on topical use, as suggested in the future perspective section. These approaches could circumvent the systemic metabolic disturbances associated with oral antifungal administration while leveraging known pharmacokinetic and safety profiles from psychiatric applications. Therefore, sertraline is a mechanistically informative but clinically challenging antifungal candidate.

## Data Availability

No new data was created in this work.
